# Circuital characterisation of space-charge motion with a time-varying applied bias

**DOI:** 10.1038/srep11738

**Published:** 2015-07-02

**Authors:** Chul Kim, Eun-Yi Moon, Jungho Hwang, Hiki Hong

**Affiliations:** 1 School of Mechanical Engineering, Yonsei University, Seoul 120-749, Republic of Korea; 2 Department of Bioscience and Biotechnology, Sejong University, Seoul 143-747, Republic of Korea; 3 Department of Mechanical Engineering, Kyung Hee University, Yongin 446-701, Republic of Korea

## Abstract

Understanding the behaviour of space-charge between two electrodes is important for a number of applications. The Shockley-Ramo theorem and equivalent circuit models are useful for this; however, fundamental questions of the microscopic nature of the space-charge remain, including the meaning of capacitance and its evolution into a bulk property. Here we show that the microscopic details of the space-charge in terms of resistance and capacitance evolve in a parallel topology to give the macroscopic behaviour via a charge-based circuit or electric-field-based circuit. We describe two approaches to this problem, both of which are based on energy conservation: the energy-to-current transformation rule, and an energy-equivalence-based definition of capacitance. We identify a significant capacitive current due to the rate of change of the capacitance. Further analysis shows that Shockley-Ramo theorem does not apply with a time-varying applied bias, and an additional electric-field-based current is identified to describe the resulting motion of the space-charge. Our results and approach provide a facile platform for a comprehensive understanding of the behaviour of space-charge between electrodes.

An external current may be induced by the motion of space-charge between two electrodes. The Shockley-Ramo theorem^1^,^2^ is widely acknowledged to provide a basic description of the external current. This theorem describes the current induced by a moving point space-charge between electrodes. It has a wide range of applications, including biological ion channels^3^,^4^,^5^, solid-state devices^6^,^7^,^8^, electrochemistry^9^,^10^, electrical discharge^11^,^12^, and semiconductor detectors^13^,^14^,^15^. The Shockley-Ramo theorem is useful for understanding the behaviour of point space-charges. For example, when this theorem is applied to calculate the gating current of an ion channel, it relates the microscopic motion of the ions to the macroscopic current recorded using a voltage clamp^3^. The Shockley-Ramo theorem does not, however, provide circuital information on the induced current. Although the motion of space-charge has aspects of resistance and capacitance, it provides only one combined current.

An equivalent circuit approach to understanding the space-charge behaviour may be more useful for investigation of dielectric barrier discharge^16^,^17^,^18^. Equivalent circuit models have been employed in nanopore sequencing^19^,^20^,^21^ and nano-scale devices^22^,^23^,^24^. Such circuital studies predetermine the overall equivalent circuit, and the circuit components are typically evaluated experimentally. The resulting capacitance can be fixed^20^,^21^ or time-varying^16^,^17^,^18^,^19^; however, the physical relationship between the space-charge and equivalent capacitance is not well understood. Nanopore studies have posed some basic and general questions, including the meaning of capacitance, and its evolution into the corresponding bulk properties^25^.

Here we study the microscopic motion of discrete charges in circuital terms, and attempt to characterise the evolution thereof into macroscopic circuit components. One restriction in the derivation of the Shockley-Ramo theorem is the fixed applied voltage between the electrodes^1^,^2^. With our analysis, however, we consider a time-varying applied voltage for a simple system composed of only positive electrode-charge and positive space-charge. Our framework is based on conservation of the electrostatic potential energy of the system. This leads to two approaches: the energy-to-current transformation rule and the energy-equivalence-based definition of capacitance. Results obtained using this theoretical framework are validated via a comparison between experimental and numerical analyses of an air corona discharge with a time-varying applied voltage; i.e., a sinusoidally varying bias with a DC offset. Additional analyses are carried out to compare our results with those of the Shockley-Ramo theorem and the electric current that results from applying the Ampère-Maxwell equation. In particular, the time dependence of the capacitance due to space-charge is examined in detail.

## Results

### Framework

**Figure 1:**
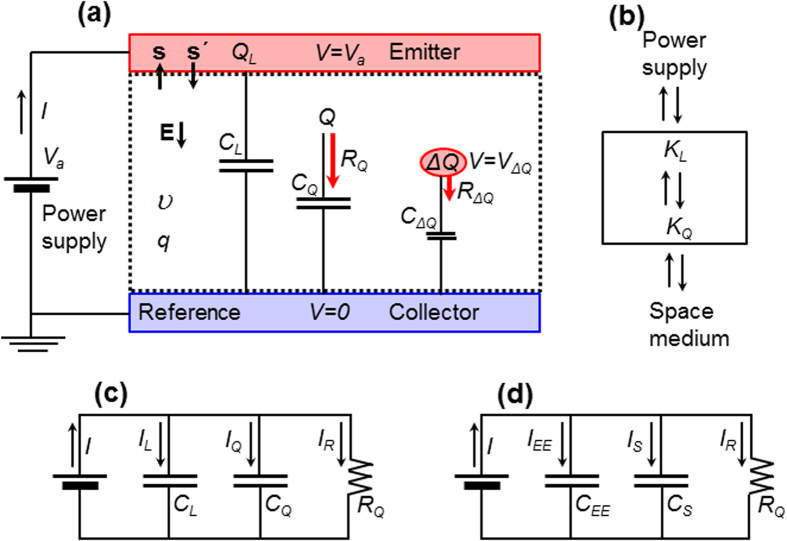
Schematic diagram of the two-electrode system and corresponding circuit diagrams. (**a**) The two-electrode configuration, where *Q*_*L*_ is charge on the electrode, 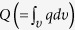 is the total space-charge, *υ* is the volume enclosed by dotted line, *C*_*L*_ is the capacitance between emitter and collector, *C*_*Q*_ is the equivalent capacitance of the charge *Q*, *R*_*Q*_ is the equivalent resistance of the charge *Q*, *C*_Δ*Q*_ is the equivalent capacitance of Δ*Q*, *R*_Δ*Q*_ is the equivalent resistance of Δ*Q*, *V*_Δ*Q*_ is the local electric potential imposed on Δ*Q*, **s** and **s′** are vector areas, and **E**↓ is the downward electric field. The downward field causes space-charge to drift downwards, as shown by the red downward arrows (↓). (**b)** A schematic diagram of the energy interactions. The rectangle shows the boundary of the system. (**c)** The equivalent electric circuit with charge-based current continuity. (**d)** The equivalent electric circuit with electric-field-based current continuity.

The proposed system consists of two electrodes separated by space, as shown in Fig. 1a. A positive electrode (the emitter) is connected to the positive terminal of a power supply, and the negative electrode (the collector) is grounded. The applied bias between the electrodes is *V*_*a*_ and the external current is *I*. The collector is set as a reference for the electric potential, so that the potential of the collector is zero and that of the emitter is *V*_*a*_. The system consists of a positive electrode-charge *Q*_*L*_ and a positive space-charge *Q*. The electrostatic potential energy due to *Q*_*L*_ can be expressed as *K*_*L*_ = 1/2*Q*_*L*_*V*_*a*_, and the electrostatic potential energy due to *Q* is given by 
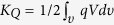
, where *υ* is the volume between the electrodes, *q* is the charge density, and *V* is the electric potential with respect to the reference^26^. The quantity *K*_*Q*_ can be transformed in terms of the electric field using integration by parts after applying Gauss’s law, and assuming that a constant permittivity *ε*^26^; i.e.,


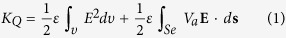


where *E*^2^ = **E** · **E**, **E**( = −∇*V*) is the electric field, *Se* is the surface of the emitter, and **s** is the vector area (see Fig. 1a). The above relation can be written as *K*_*Q*_ = *K*_*EE*_ + *K*_*VE*_, where  
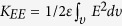
 and 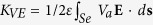. We use the symbol *K* to represent the charge-based electrostatic potential energy; i.e., *K*_*L*_ and *K*_*Q*_, and we represent the electric-field-based electrical energy as *K*_*EE*_ and *K*_*VE*_.

Figure 1b shows the total energy of the system. We use the same assumption as that used in the Shockley-Ramo theorem; i.e., that magnetic effects are negligible in the quasistatic regime^1^,^2^. The total electrical energy *K*_*M*_ in the system is then the sum of *K*_*L*_ and *K*_*Q*_ (i.e., *K*_*M*_ = *K*_*L*_ + *K*_*Q*_). Consider the case whereby the power supply provides the system with an electric power *P*(=*IV*_*a*_) and there is no energy interaction with the space medium. Using conservation of energy, this input power will increase *K*_*M*_ to satisfy the relation 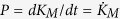. Therefore, if we know 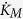, the external circuit current can be expressed as 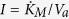. Based on this rationale, here we implement a streamlined energy-to-current transformation rule such that *K*_*X*_ (the electrical energy) → 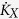
(=*dK*_*X*_/*dt*, the equivalent circuit power) → *I*_*X*_(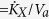, the equivalent circuit current), where the subscript *X* represents a specific characteristic. Using this rule, we can conveniently transform the foregoing electrical energies to equivalent circuit currents for a given applied voltage such that *K*_*L*_ → 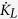 → *I*_*L*_, *K*_*Q*_ → 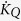 → *I*_*Q*_, *K*_*EE*_ → 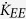 → *I*_*EE*_, and *K*_*VE*_ → 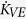 → *I*_*VE*_. In the same manner, energy relations are readily transformed to current relations such that *K*_*Q*_ = *K*_*EE*_ + *K*_*VE*_ → 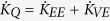 → *I*_*Q*_ = *I*_*EE*_ + *I*_*VE*_ and *K*_*M*_ = *K*_*L*_ + *K*_*Q*_ → 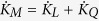 → *I*_*M*_ = *I*_*L*_ + *I*_*Q*_.

The capacitance between the two electrodes is usually defined as *Q*_*L*_ = *C*_*L*_*V*_*a*_, where *C*_*L*_ is the capacitance of the electrodes (see Fig. 1a), and is the constant of proportionality between *Q*_*L*_ and *V*_*a*_^26^. Here *C*_*L*_ is dependent only on the geometry of the system and the permittivity; *C*_*L*_ = *ε*(*A*/*d*_*g*_) for a pair of parallel electrodes, where *A* is the area of the electrodes and *d*_*g*_ is the separation between them. Here we assume that the electrodes are perfect electric conductors, so that the electric field inside the conductor is zero^26^ and the electric potential of the electrode is equal to the applied bias. In addition, no external field can penetrate the conductor and the net induced charge is zero^26^. It follows that the electric field generated by the space-charge should not change the charge on the electrode *Q*_*L*_, and that *C*_*L*_ is constant, even in the presence of space-charge. We define the capacitance using the following energy equivalence relation^26^:


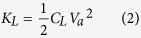


If we apply *K*_*L*_ = 1/2*Q*_*L*_*V*_*a*_, the above expression reduces to *Q*_*L*_ = *C*_*L*_*V*_*a*_; however, equation (2) is of interest because our analysis is based on conservation of energy. For example, the capacitance due to the total space-charge (i.e., *C*_*Q*_ in Fig. 1a) is defined by 
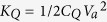
. Furthermore, this method can be extended to the electrical energy which does not include charge. The capacitance of the electrical energy stored in the electric field can be defined from 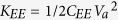 22. Note that the above two definitions of capacitance result in different currents when the system is time varying. This will be discussed in greater detail later.

### Discrete circuit components and evolution

As shown in Fig. 1a, a discrete positive charge Δ*Q* drifting between the two electrodes is assumed to have a discrete resistance *R*_Δ*Q*_. As Δ*Q* drifts due to the Coulomb force **F**_Δ*Q*_(=Δ*Q***E**), it does resistive Ohmic work due to ion-neutral collisions, where the work rate is **F**_Δ*Q*_ · **U**_Δ*Q*_^27^. Here **U**_Δ*Q*_(=*μ***E**) is the velocity of the discrete charge Δ*Q* and *μ* is the electrical mobility. In this way, the electrical energy of the system is converted into the thermal energy in the space between the electrodes^27^. The notation 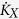 can be used to describe the Ohmic work done by the charge Δ*Q* because this energy dissipation process consumes electrostatic potential energy. This microscopic process can be described by the discrete energy dissipation rate 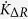, which satisfies the energy equivalence relation 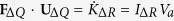, where *I*_Δ*R*_ is the equivalent discrete circuit current of *R*_Δ*Q*_. From this relation and using Ohm’s law (i.e., *V*_*a*_ = *I*_Δ*R*_*R*_Δ*Q*_), we find that the discrete circuit resistance *R*_Δ*Q*_ is given by


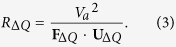


The total Ohmic work rate 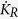
(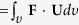, **F** = *q***E**, **U** = *μ***E**)^27^ in the space between the electrodes and the corresponding circuit resistance *R*_*Q*_ (see Fig. 1a) should satisfy the power relation 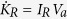 as well as Ohm’s law *V*_*a*_ = *I*_*R*_*R*_*Q*_, where *I*_*R*_ is the equivalent circuit current of *R*_*Q*_. Using conservation of energy, the total 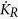 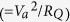 is the sum of each discrete 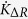 (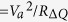, see equation (3)), which leads to the relation 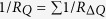. Therefore, all of the discrete resistances can be said to evolve in parallel into the equivalent circuit resistance.

Note that Δ*Q* is assumed to have a discrete capacitance *C*_Δ*Q*_, as shown in Fig. 1a. Because the charge Δ*Q* leads to a local electric potential *V*_Δ*Q*_, the electrostatic potential energy due to Δ*Q* can be expressed as *K*_Δ*Q*_ = 1/2Δ*QV*_Δ*Q*_. From equation (2), we may write 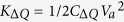 to define *C*_Δ*Q*_ as the equivalent discrete circuit capacitance due to Δ*Q*. From those two expressions, we may write *C*_Δ*Q*_ as follows:


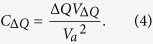


From conservation of energy, the total energy 
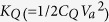
 is the sum of each discrete term 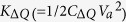, which leads to 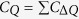. Hence, all of the discrete capacitances can also be said to evolve into the equivalent circuit capacitance in a parallel topology.

### Development of the charge-based circuit

The overall instantaneous application of conservation of energy for the system leads to 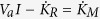, where *V*_*a*_*I* is the input power from the power supply, 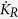 is the power loss from the system to the space medium, and 
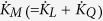
 is the rate of change of the total electrostatic potential energy inside the system. This in turn leads to 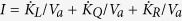, after dividing both sides by *V*_*a*_, and we arrive at the following charge-based current continuity equation:


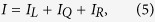


where *I*_*L*_ and *I*_*Q*_ are the capacitive contributions to the currents, which come from the non-dissipative electrostatic potential energies, whereas *I*_*R*_ is a resistive current. Therefore, the above charge-based current continuity relation can be represented as a parallel circuit composed of two capacitors *C*_*L*_ and *C*_*Q*_, and one resistor *R*_*Q*_, as shown in Fig. 1c.

### Transformation to an electric-field-based circuit

The currents *I*_*L*_ and *I*_*Q*_ are transformed from *K*_*L*_ and *K*_*Q*_, respectively. To understand the current continuity in terms of the electric field, *K*_*L*_ and *K*_*Q*_ should be transformed from the charge-based electrostatic potential energies to the electric-field-based currents. As we have already shown, *K*_*Q*_ is transformed using the current relation *I*_*Q*_ = *I*_*EE*_ + *I*_*VE*_. In addition, *K*_*L*_ can be transformed into the electric field energy. If we assume the surface of the emitter is a Gaussian surface, Gauss’s law for the emitter charge *Q*_*L*_ can be written as 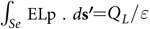 when there is no space-charge; here **E**_*Lp*_ is the Laplacian electric field and **s′** is the vector area (**s′** = − **s**, see Fig. 1a). This relation however applies even in the presence of space-charge, since *Q*_*L*_ is not influenced by the space-charge, as discussed previously. By substituting the expression 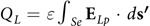 into *K*_*L*_ = 1/2*Q*_*L*_*V*_*a*_, we obtain 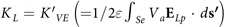, as well as 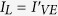 by employing the transformation rule. By inserting *I*_*Q*_ = *I*_*EE*_ + *I*_*VE*_ and 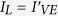 into equation (5), we obtain 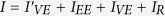. Consequently, if 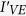 and *I*_*VE*_ are combined into an equivalent current 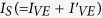, we arrive at the following electric-field-based current continuity:


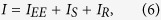


where *I*_*S*_ given by


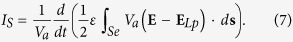


The term *I*_*EE*_ in equation (6) is the non-dissipative volumetric current, and includes a volume integral (see *K*_*EE*_ in equation (1)), whereas *I*_*S*_ in equation (7) is a non-dissipative surficial current which includes the surface integral. The volumetric current *I*_*EE*_ and surficial current *I*_*S*_ are geometrically independent. Hence, the above electric-field-based current continuity relation can be represented as a parallel circuit composed of two capacitors *C*_*EE*_ and *C*_*S*_, and one resistor *R*_*Q*_, as shown in Fig. 1d.

### Validation

**Figure 2:**
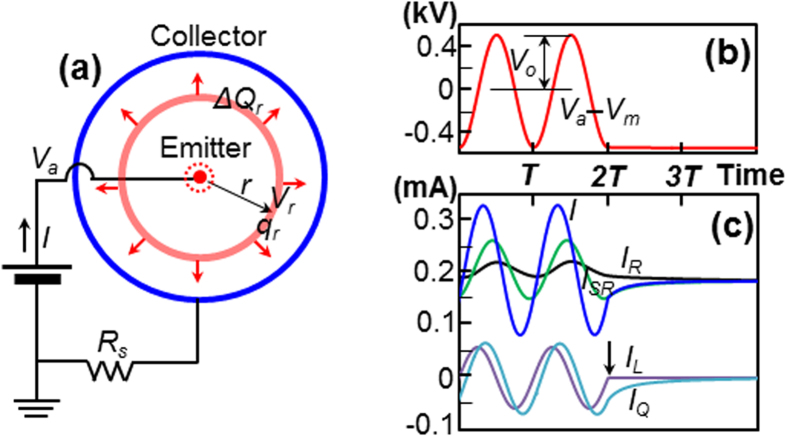
Experimental setup and simulated currents. (**a**) A schematic diagram showing the wire-to-cylinder positive air corona discharge. The outer radius of the emitter was *r*_*e*_ = 0.02 mm, the inner radius of the collector was *r*_*c*_ = 17 mm, and the length (perpendicular depth of cross-section) was *l* = 100 mm. The theoretical value of *C*_*L*_ is given by 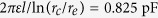, where *ε* is the permittivity of free space and *R*_*S*_ is the shunt resistance. The eight radial arrows show the axisymmetric drift of annular discrete charge Δ*Q*_*r*_ at radius *r*. *V*_*r*_ is the electric potential at *r* and *q*_*r*_ is the charge density at *r*. The dotted circle shows the boundary of the plasma sheath with the radius *r*_*p*_ ≈ 3*r*_*e*_(see Methods). (**b**) The waveform of applied voltage used for experiments and simulation (the left half shows *t* ≤ 2*T*), where *V*_*m*_ = 9 kV, *V*_*o*_ = 0.53 kV, *f* = 20 kHz and *T* = 50 μs. (**c**) Transition of the simulated currents from time-varying to the steady-state. The current *I* is shown in blue, *I*_*SR*_ in green, and *I*_*L*_ = 0 is shown by the symbol ↓. The left-hand side corresponds to the loop *V*_*a*_-*I* (see Fig. 4a).

An experimental setup consisting of an axisymmetric wire-to-cylinder positive air corona discharge^28^ was used to validate the theoretical framework described here, as shown in Fig. 2a (see Methods for details of the experiment). If a positive voltage applied to the emitter exceeds the corona discharge initiation voltage, a positive charge (i.e., a collection of positive ions) will be generated in a thin plasma sheath around the emitter^28^,^29^ (shown by the dotted circle in Fig. 2a), and that charge will drift toward the collector due to the Coulomb force^28^,^29^. Here we assume that the applied bias is described by a sinusoidal waveform with a DC offset, as shown in the leftmost two waveforms in Fig. 2b, i.e., *V*_*a*_ = *V*_*m*_ + *V*_*o*_ sin (2*πf*)*t*, where *V*_*m*_ is the mean voltage (i.e., DC offset), *V*_*o*_ is the amplitude of the time-varying component, *f* is the frequency of the time varying component (so that *T* = 1/*f* is the period), and *t* is time. The purpose of this waveform is to enable time-varying motion of the space-charge.

**Figure 3:**
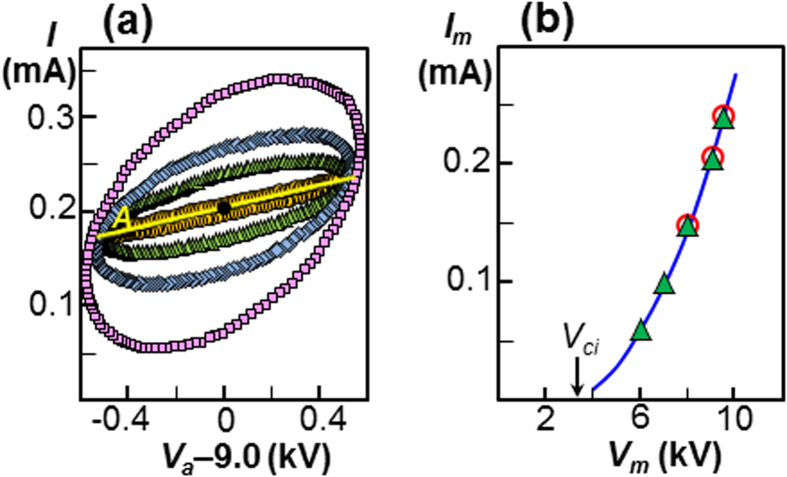
Experimental validation of the parallel circuit topology. (**a**) *V*_*a*_-*I* loops for frequencies of 20 kHz (□), 10 kHz (◊) 5 kHz (△) and 1 kHz (○). The coincidences of *I*_*mean*_(•) were as follows: 0.206 mA for 0.2 kHz, 0.207 mA for 0.5 kHz, 0.206 mA for 1 kHz, 0.204 mA for 2 kHz, 0.207 mA for 5 kHz, 0.204 mA for 10 kHz and 0.204 mA for 20 kHz, average: 0.2054 mA, standard deviation: 0.0013 mA. The 2-kHz loop is plotted between 1- and 5-kHz loops (not shown). The 0.2-kHz and 0.5-kHz loops were almost identical to line *A*. (**b**) The experimental DC current-voltage curve reveals typical wire-to-cylinder corona discharge, and can be fitted with *I* = 0.00405 *V*_*a*_(*V*_*a*_ − *V*_*ci*_), where *V*_*ci*_ = 3.4 kV is the corona discharge initiation voltage^28^. Data for *f* = 20 kHz and *V*_*o*_ = 1.0 kV are shown by the ‘○’ symbols and data for *f* = 100 kHz and *V*_*o*_ = 0.1 kV by the ‘▲’ symbols. Difference between DC data and 8 symbols; average: 0.00042 mA, standard deviation: 0.0012 mA.

First, we attempt to reproduce the parallel topology discussed above. The terms *C*_*L*_ and *C*_*Q*_ (see Fig. 1c) result from *K*_*L*_ (due to the electrodes) and *K*_*Q*_ (due to the space-charge), whereas *C*_*EE*_ and *C*_*S*_ (see Fig. 1d) result from *K*_*EE*_ (for the volume) and *K*_*S*_ (for the surface). The summation of these geometrically separated energies corresponds to the parallel connection of two capacitors. However, concerning the space-charge, it is not clear whether the drift of the point charge Δ*Q* (see Fig. 1a) corresponds to a parallel or series connection of *C*_Δ*Q*_ and *R*_Δ*Q*_. In this respect, it is helpful to experimentally confirm the parallel connection between *R*_*Q*_ and the two capacitances. With periodic conditions, the mean circuit current 
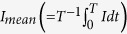
 of the proposed parallel circuit should always be equal to the mean resistor current for a given *V*_*m*_ because the mean of the capacitor current is zero regardless of frequency. For *V*_*m*_ = 9 kV, as shown in Fig. 3a, the experimental result reveals that the *V*_*a*_-*I* loop contracted to the line *A* as the frequency decreased from 20 kHz to 1 kHz. Consequently, *I*_*mean*_ for each axisymmetric current loop exactly coincides with one centred point for all frequencies, which corresponds to a parallel circuit topology. Figure 3b provides further support of this when *V*_*m*_ and *V*_*o*_ are varied. The solid line in Fig. 3b shows the DC current-voltage curve, which is the same as the *V*_*m*_-*I*_*mean*_ curve for *f* = 0 kHz. For *f* = 20 kHz and *V*_*o*_ = 1.0 kV, the centred point coincides exactly with the *V*_*m*_-*I*_*mean*_ curve at the three points of *V*_*m*_, and with *f* = 100 kHz and *V*_*o*_ = 0.1 kV, the centred point coincides exactly with the *V*_*m*_-*I*_*mean*_ curve at the five points of *V*_*m*_.

**Figure 4:**
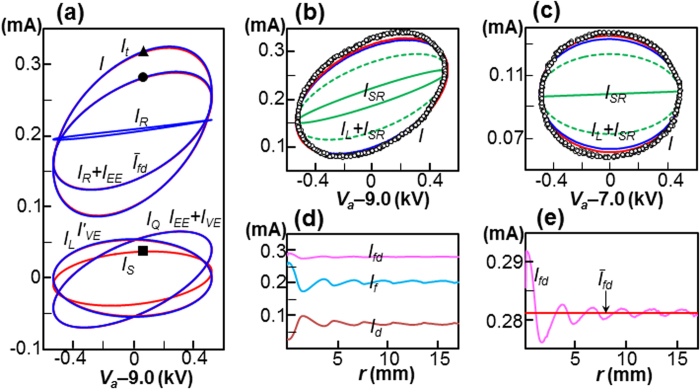
Validation of the theoretical work. (**a**) Simulated *V*_*a*_-*I* loops for *f* = 20 kHz, *V*_*m*_ = 9.0 kV and *V*_*o*_ = 0.53 kV. The components of the current *I*, *I*_*R*_, *I*_*R*_ + *I*_*EE*_, *I*_*L*_ and *I*_*Q*_ are shown in blue, and *I*_*t*_, 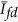, *I*′_*VE*_, *I*_*EE*_ + *I*_*VE*_ and *I*_*S*_ are shown in red. The coincidences between *I* and *I*_*t*_, *I*_*R*_ + *I*_*EE*_ and 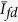, *I*_*L*_ and 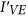, *I*_*Q*_ and *I*_*EE*_ + *I*_*VE*_ were so good that the differences cannot be distinguished. ▲: *I*(0.319 mA), •: *I*_*R*_ + *I*_*EE*_ (0.282 mA), ■: *I*_*S*_(0.037 mA). (**b**) Circuit current comparison between experiment (○) and simulation (shown by the blue inner loop) for the same conditions as Fig. 4a. The measured capacitance (*C*_*L*_ = 0.89 pF) is shown by the red outer loop. The current *I*_*SR*_ is shown by the solid green curve, and *I*_*L*_ + *I*_*SR*_ is shown by the dashed green curve. (**c**) Additional comparison between experiment (○) and simulation for *f* = 100 kHz, *V*_*m*_ = 7.0 kV and *V*_*o*_ = 0.05 kV. The current *I*_*SR*_ is shown by the solid green curve, and *I*_*L*_ + *I*_*SR*_ is shown by the dashed green curve. (**d**) Instantaneous profiles of *I*_*f*_ and *I*_*d*_ plotted with as a function of the radius for *r*_*e*_ ≤ *r* ≤ *r*_*c*_. (**e**) A magnified profile of *I*_*fd*_ shown in (**d**).

To quantitatively understand the theoretical framework described above, the Poisson and charge conservation equations were solved for the experimental setup to obtain the time-varying equivalent currents and circuit components (see Methods for details of the simulation). Note that the electrode current *I*_*L*_ was calculated from 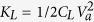 using the theoretical value of *C*_*L*_ (0.825 pF, see Fig. 2a). Figure 4a shows various current-voltage loops for *f* = 20 kHz, which are plotted for the fully periodic state. In the lower part of the figure, the loop *I*_*Q*_ coincides with the loop *I*_*EE*_ + *I*_*VE*_, whereas the loop *I*_*L*_ coincides with the loop 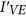. This supports the relations *I*_*Q*_ = *I*_*EE*_ + *I*_*VE*_ and 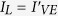, which are pivotal in the charge-to-field transformation. In addition, the surficial current *I*_*S*_ is revealed to be a considerable loop. The complete coincidence of the loop *I* (i.e., the sum of *I*_*L*_, *I*_*Q*_ and *I*_*R*_) and the loop *I*_*t*_ (the sum of *I*_*EE*_, *I*_*S*_ and *I*_*R*_) supports the equivalence of charge-based and electric-field-based approaches.

Figure 4b shows a comparison between the measured circuit current *I* and the simulated current for the case shown in Fig. 4a. Although the two loops are in very good agreement, both in terms of the overall shape and the instantaneous data, a small discrepancy was observed at the top. The experimentally measured loop was somewhat distorted, compared with the simulated data, which is attributed to the incomplete sine wave generated by the power supply, and the measured capacitance *C*_*L*_ = 0.89 pF was found to provide better agreement. As shown in Fig. 4c, similar agreement was achieved with *f* = 100 kHz, which supports our theoretical result; i.e., the two equivalent circuits shown in Fig. 1.

### Comparison with the Shockley-Ramo theorem

This theorem expresses the circuit current induced by the point space-charge Δ*Q* as Δ*Q***U**_Δ*Q*_ · **E**_*Lp*_ for an applied bias of 1 V, which leads to the relation 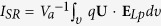 30 for the total space-charge. When we plot *I*_*SR*_, as shown in Fig. 4b,c the results were in poor agreement with the experimentally measured current for both cases. It has been reported^1^,^13^,^31^ that the electrode current *I*_*L*_ should be added to *I*_*SR*_ to provide the correct external circuit current, since *I*_*SR*_ is obtained with a fixed *V*_*a*_. This appears reasonable for a time-varying *V*_*a*_; however, as shown in Fig. 4b,c, *I*_*L*_ + *I*_*SR*_ did not provide good agreement with the experimentally measured current. Figure 2c shows the transition of the current from the time-varying applied bias to the steady-state. The current *I* merged with *I*_*SR*_ as *V*_*a*_ became constant, and finally coincided with *I*_*R*_ in the steady state as *I*_*Q*_ became zero. This result suggests that *I*_*SR*_ does not predict the circuit current for the motion of space-charge when the applied voltage varies with time.

### Comparison with spatial electric currents

The currents shown in the charge-based and electric-field-based current continuities are the equivalent circuit currents, rather than actual currents that flow in the space between two electrodes. Here we compare the equivalent circuit currents with the spatial current density **J** that appears in Ampère-Maxwell equation^26^, i.e., ∇ × **H** = **J**, where **H** is the magnetic field, and **J** = **J**_*f*_ + **J**_*d*_, where **J**_*f*_ ( = *qU*) is the conduction current density of free charge and **J**_*d*_ ( = *ε*∂**E**/∂*t*) is the displacement current density. To understand how **J** relates to the circuit current, we attempt to visualise the spatial **J**_*f*_ and **J**_*d*_ at a moment, which corresponds to the symbols ▲, • and ■ shown in Fig. 4a. Figure 4d shows the radial distributions of the free charge current *I*_*f*_ (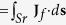, where *Sr* is surface at radius *r*; see Fig. 2a,) and displacement current 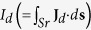. The ripples that appear in *I*_*f*_ and *I*_*d*_ are due to fluctuations in the applied voltage. Although the shape of *I*_*fd*_ ( = *I*_*f*_  + *I*_*d*_) appears to be flat with low ripples because the fluctuations of *I*_*f*_ and *I*_*d*_ cancel, those ripples exhibit a wave pattern, as shown by the magnified plot in Fig. 4e. Let 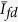 be the volume average of *I*_*fd*_; although, we find agreement between 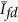 (0.281 mA) and *I*_*R*_ + *I*_*EE*_ (0.282 mA), this does not imply an exact equivalence of 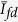 and *I*_*R*_ + *I*_*EE*_, and rather suggests that the **J** is linked to the volumetric terms *I*_*R*_ + *I*_*EE*_. Subsequently, the agreement between *I* (0.319 mA) and 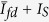 (0.318 mA, where *I*_*S*_ = 0.037 mA) supports our theoretical identification of surficial current *I*_*S*_. Figure 4a shows further overall agreement between 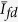 and *I*_*R*_ + *I*_*EE*_ for the entire loop.

The numerically demonstrated link between the spatially averaged 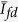 and the volumetric *I*_*R*_ + *I*_*EE*_ is supported theoretically. The term 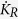 can be re-written as 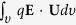, whereas *K*_*EE*_ in equation (1) is differentiated to give 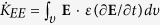. Consequently, 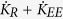 leads to 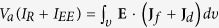. If we resort to the definition of work rate **E** · **J**_*f*_^26^, which is done by the moving charge, the above relation suggests that **J**_*f*_ is linked to *I*_*R*_ and **J**_*d*_ is linked to *I*_*EE*_.

### Evaluation of circuit components

**Figure 5:**
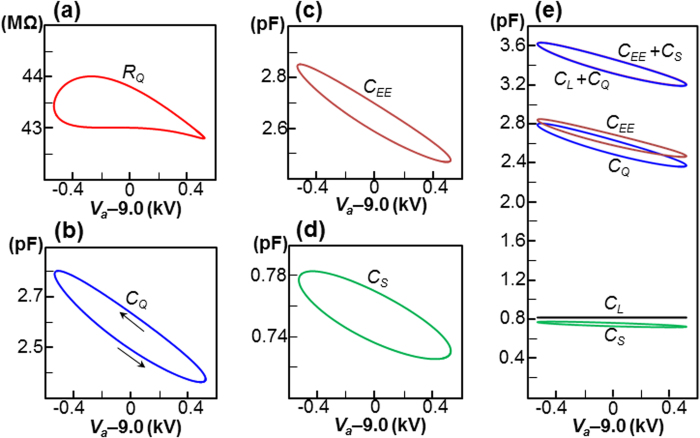
Evaluation of time-varying circuit components at 20 kHz. (**a**) *V*_*a*_-*R*_*Q*_. (**b**) *V*_*a*_-*C*_*Q*_. The direction of the two arrows agrees with the time increment. (**c**) *V*_*a*_-*C*_*EE*_. (**d**) *V*_*a*_-*C*_*S*_. (**e**) All capacitances are compared. The *C*_*L*_ + *C*_*Q*_ loop (blue) coincides with *C*_*EE*_ + *C*_*S*_ loop (red) such that the difference is barely distinguishable.

There are five circuit components: *C*_*L*_, *C*_*Q*_, *R*_*Q*_, *C*_*EE*_ and *C*_*S*_ in the two proposed circuits. With the exception of *C*_*L*_, the other four components can be calculated from the acquired *V* and *q* fields. The resistance *R*_*Q*_ can be calculated using equation (3), whereas three capacitances are calculated using equation (2); we arrive at the following expressions: 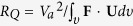, 
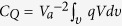
, 
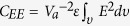
 and 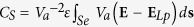. With the same conditions as the data shown in Fig. 4a, the four components are plotted in Fig. 5a–d. Interestingly, *R*_*Q*_ was not fixed. This quantity forms a pinched loop that relates to the time-varying characteristics of the circuit in a similar manner to a memristor *R*_*m*_^32^, and is identical to *R*_*Q*_ from the definition of *V*_*a*_ = *I*_*R*_*R*_*m*_. The three capacitances *C*_*Q*_, *C*_*EE*_ and *C*_*S*_ lead to three elliptical loops that describe the time-varying characteristics of the system. All of the capacitances, including *C*_*L*_, are compared in Fig. 5e. The two parallel charge-based and electric-field-based circuits shown in Fig. 1 are equivalent, so that *C*_*L*_ + *C*_*Q*_ should be equal to *C*_*EE*_ + *C*_*S*_. The data plotted in Fig. 5e reveal a complete coincidence between *C*_*L*_ + *C*_*Q*_ and *C*_*EE*_ + *C*_*S*_. The data plotted in Fig. 5e show that this is indeed the case. This agreement confirms the effectiveness of the proposed method to define the capacitance, which can be extended to the electric field energy.

### Capacitive current and time-varying capacitance

We examine the current flowing in the time-varying capacitance *C*_*Q*_, as shown in Fig. 5b. The time-varying capacitance (which is similar to a memcapacitor^19^,^32^,^33^) is defined as *Q* = *C*_*Q*_*V*_*a*_, where the capacitor current *I*_*QA*_ is defined by applying the product rule for differentiation to *I*_*QA*_ = *dQ*/*dt* = *d*(*C*_*Q*_*V*_*a*_)/*dt*^32^; i.e.,


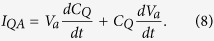


**Figure 6:**
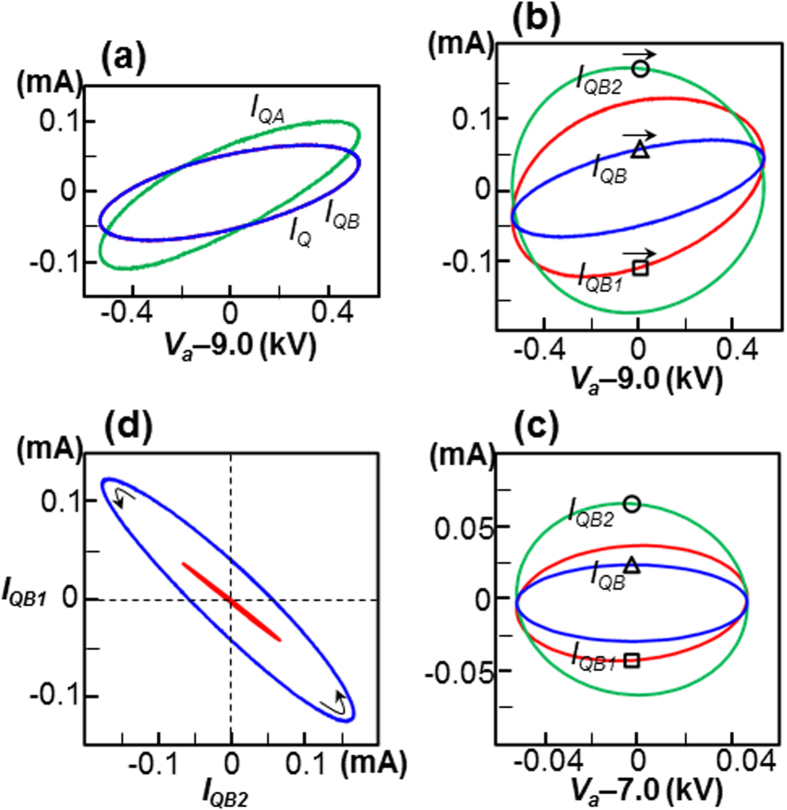
Analysis of capacitive current due to space-charge. (**a**) Comparison between *I*_*QA*_ and *I*_*QB*_ for 20 kHz (see Fig. 4a). The *I*_*Q*_ loop (blue) coincides with the *I*_*QB*_ loop (red) such that the difference is not clearly distinguishable. (**b**) The quantity *I*_*QB*_ (△ symbol) plotted in Fig. 6a is decomposed into *I*_*QB*1_ (□ symbol) and *I*_*QB*2_ (○ symbol). The directions of the three arrows agree with the time increment. (**c**) The *I*_*QB*_ (△ symbol) is decomposed into *I*_*QB*1_ (□ symbol) and *I*_*QB*2_ (○ symbol) for *f* = 100 kHz (see Fig. 4c). (**d**) *I*_*QB*1_ and *I*_*QB*2_ relations; the outer loop (blue) corresponds to *f* = 20 kHz and the thin inner loop (red) to *f* = 100 kHz. The direction of the two arrows agrees with the time increment.

The capacitor current *I*_*QB*_, which results from the energy equivalence of 
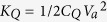
, can be expressed by applying the product rule to 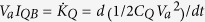; i.e.,


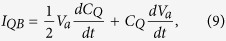


where *I*_*QA*_ and *I*_*QB*_ reveal an apparent difference in the first term, which reflects the effects of the unsteady capacitance; i.e., *V*_*a*_(*dC*_*Q*_/*dt*) for *I*_*QA*_, and 1/2*V*_*a*_(*dC*_*Q*_/*dt*) for *I*_*QB*_. It should be noted, however, that both *I*_*QA*_ and *I*_*QB*_ reduce to the usual capacitor current relation of *I*_*Q*_ = *C*_*Q*_(*dV*_*a*_/*dt*) when *C*_*Q*_ is constant. Figure 6a shows a comparison between *I*_*QA*_ and *I*_*QB*_ for *f* = 20 kHz. The exact coincidence of *I*_*QB*_ with *I*_*Q*_, which is calculated from 
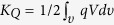
, suggests that equation (9) is more plausible than equation (8) to describe the time-varying capacitance.

Figure 5e shows that the electrode capacitance *C*_*L*_ is approximately one third of *C*_*Q*_. It follows that *I*_*L*_ should be one third of *I*_*Q*_, according to the usual capacitor current relation; however, the *I*_*Q*_ and *I*_*L*_ loops plotted in Fig. 4a have approximately same magnitude at the point *V*_*a*_ ≈ 9 kV. This apparent contradiction can be resolved by considering the effect of the time-varying capacitance. In Fig. 6b, the quantity *I*_*QB*_ that is plotted in Fig. 6a is decomposed into that due to the time-varying capacitance, which is denoted *I*_*QB*1_ ( = 1/2*V*_*a*_(*dC*_*Q*_/*dt*)), and that due to the steady-state capacitance, denoted *I*_*QB*2_ ( = *C*_*Q*_(*dV*_*a*_/*dt*)), where *I*_*QB*_ = *I*_*QB*1_ + *I*_*QB*2_. With *V*_*a*_ ≈ 9 kV, the relatively large value of *I*_*QB*2_ (0.165 mA) was counterbalanced by the negative value of *I*_*QB*1_ (−0.113 mA) to create a relatively small value of *I*_*QB*_ (0.053 mA). This counterbalancing effect of the time-varying capacitance is sufficiently large (*I*_*QB*1_/*I*_*QB*2_ = −0.68 ≈ −2/3) to allow *I*_*QB*_ to be one third of the usual capacitor current *I*_*QB*2_. With *f* = 100 kHz, as shown in Fig. 6c, a similar counterbalancing effect was observed at the point *V*_*a*_ ≈ 7 kV; i.e., *I*_*QB*2_ = 0.066 mA, *I*_*QB*1_ = −0.044 mA and *I*_*QB*_ = 0.022 mA, so that we have *I*_*QB*1_/*I*_*QB*2_ = −0.67. As shown in Fig. 6d, the *I*_*QB*2_-*I*_*QB*1_ relations form elliptical loops, with a narrow outer loop for *f* = 20 kHz and a thin inner loop for *f* = 100 kHz. These two points correspond to the bottom-right corners of the two loops. The lower turning point shown in Fig. 6d corresponds to the arrow to the right (→) in Fig. 6b and the lower arrow (↘) of Fig. 5b. This tilted arrow corresponds to an increase in *V*_*a*_ (*dV*_*a*_/*dt* > 0) and decrease in *C*_*Q*_ (*dC*_*Q*_/*dt* < 0), which leads to a positive *I*_*QB*2_ and a negative *I*_*QB*1_ at the lower turning point of Fig. 6d. In the same manner, the upper turning point shown in Fig. 6d corresponds with the upper arrow (↖) of Fig. 5b. This tilted arrow corresponds to a decrease in *V*_*a*_ (*dV*_*a*_/*dt* < 0) and an increase in *C*_*Q*_ (*dC*_*Q*_/*dt* > 0), which gives a negative *I*_*QB*2_ and a positive *I*_*QB*1_ in the upper turning point of Fig. 6d.

The terms *I*_*QB*2_ and *I*_*QB*1_ exhibit a phase difference of 180° in the time domain (see Fig. 6b,c). This can be interpreted as follows: *I*_*QB*1_ behaves as an inductor current rather than the usual capacitor current *I*_*QB*2_. The overall linear relation between *I*_*QB*1_ and *I*_*QB*2_ shown in Fig. 6d is *I*_*QB*1_ ≈ −0.7*I*_*QB*2_ at both 20 kHz and 100 kHz, where the –0.7 implies a significant counterbalancing effect of the rate of change of the capacitance with the time-varying motion of the space-charge. This relation will hold only for the completely periodic case under the DC biased sinusoidal waveform of applied voltage adopted in this report.

## Discussion

Our analysis is restricted to the electrostatic potential energy of positive space-charge; however, the approach can also be applied to negative space-charge. Considering the symmetry of positive and negative charges, the reference of electric potential for the negative space-charge should be changed from collector to emitter in Fig. 1a. In this case, the electric potential energy of the discrete negative charge −Δ*Q* located in the local electric potential *V*_Δ*Q*_ is expressed as the positive value of *K*_Δ*Q*_ = 1/2(−Δ*Q*)(*V*_Δ*Q*_ − *V*_*a*_).

Regarding the method used to define the capacitance, we reconsider the usual definition of *Q*_*L*_ = *C*_*L*_*V*_*a*_ for the application to space-charge. According to this definition, the capacitance due to space-charge Δ*Q* can be deduced from 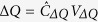, where 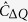 is the local capacitance for the local *V*_Δ*Q*_. The 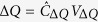 is identical to the proposed definition of 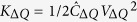 if *K*_Δ*Q*_ = 1/2Δ*QV*_Δ*Q*_; however, 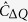 should be transformed to the circuit capacitance *C*_Δ*Q*_, which is defined at the circuital *V*_*a*_. We employ the energy equivalence relation 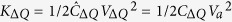 for this transformation. It follows that this transformation rule can be expressed as 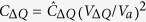, which supports our definition of the capacitance due to Δ*Q* in equation (4) when 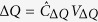 is applied. In other words, our proposed method to define the capacitance can be said to be the capacitance transformation from one voltage to another, without violating the conventional method.

The physical interpretation of the two charge-based and electric-field-based current continuities is discussed using three examples. For the time-varying case with no space-charge, the Ohmic current *I*_*R*_ due to the drift of space-charge disappears, and the surficial current *I*_*S*_ becomes zero from **E** = **E**_*Lp*_ (see equation (7)). It follows that the electric-field-based current continuity reduces to *I* = *I*_*EE*_, whereas charge-based continuity reduces to *I* = *I*_*L*_, since both *I*_*Q*_ and *I*_*R*_ are zero. The following relation *I*_*L*_ = *I*_*EE*_ can be transformed to a *K*_*L*_ = *K*_*EE*_ relation, which implies a textbook example^26^; i.e., the electrostatic potential energy stored in the electrode capacitor is equal to the volumetric electric field energy between the electrodes. In this case, the displacement current *I*_*d*_ is equal to the circuit current *I*^26^. For the steady state case with space-charge, both current continuities result in *I* = *I*_*R*_, which is an example of steady-state electrical discharge^27^,^29^,^34^. In this case, the motion of free charges, and the corresponding current *I*_*f*_ is equal to the circuit current *I*. With the time-varying case, however, as shown in Fig. 4a, the average spatial current 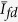 is lower than the circuit current by an amount *I*_*S*_. From the perspective of field theory, the surface integral *K*_*VE*_ in equation (1) is considered to be negligible compared with the volume integral *K*_*EE*_, by assuming a very large enclosed volume^26^. It follows that all of the electric field energy can be considered to be stored only in the spatial electric field^26^. However, *K*_*VE*_ was not neglected in the configuration discussed here because of the small enclosed volume. In this context, a so-called surficial current *I*_*S*_ may be taken as an additional spatial current.

The circuital characteristics of the motion of the space-charge with a time-varying applied bias can be summarised as follows. The microscopic behaviour of the space-charge is decomposed to a discrete equivalent circuit resistance and a discrete equivalent circuit capacitance. These microscopic components evolve in parallel with the macroscopic equivalent circuit components to form a charge-based circuit, which can be equivalently transformed into an electric-field-based circuit. All of the circuit components calculated using the space-charge and electric-potential fields vary with time, in accordance with the time-varying motion of the space-charge. With the configuration discussed here, 70% of the usual capacitive current was significantly counterbalanced by the current due to the rate of change of the capacitance. The two approaches were crucial for the theoretical framework described here: the transformation rule, which was used to transform electrical energy into an equivalent circuit current, and the method to define the capacitance, which is based on energy equivalence. The Shockley-Ramo theorem was shown to be invalid when the applied voltage was time-varying. The electric-field-based current continuity description includes an additional electric current to describe the oscillatory motion of the space-charge. We expect that our results and approach will be helpful for understanding experimental results, the design of equivalent circuits, and further theoretical studies in relevant fields.

## Methods

### Experiment

Figure 2a shows a cross-section of the wire-to-cylinder air corona discharge configuration with a central emitter formed of tungsten wire, with a diameter of 40 μm, inside a circular collector, which was formed of stainless steel pipe, with an inner diameter of 34 mm. This experimental configuration was used to approximate the one-dimensional electric potential and charge density distributions, by eliminating any edge effects. For the collector structure, five small cylindrical stainless steel tubes that were 100 mm long were assembled to form a long pipe, where for each pipe the neighbouring pipes were electrically insulated by a small air gap. The tungsten wire was placed under tension and carefully centred along the axis inside the pipes. The five collector pipes were then electrically connected in parallel, and a shunt resistance of *R*_*S*_ = 10 kΩ (see Fig. 2a) was connected to each pipe in series to measure the current. A preliminary experiment was carried out to evaluate the experimental electrode capacitance *C*_*L*_ of each pipe without space-charge (i.e., with *V*_*a*_ < *V*_*ci*_, see Fig. 3b). When *C*_*L*_ was calculated using *V*_*a*,*rms*_ = *I*_*rms*_/(*ωC*_*L*_), where *ω* = 2*πf* and the subscript *rms* stands for root-mean-square, the central pipe exhibited the smallest capacitance (0.89 pF), which corresponds to the measured *C*_*L*_. The phase difference *θ* between *V*_*a*_ and *I* was observed to change as the frequency was varied; we find *θ* = 84.2° for *f* = 20 kHz, and *θ* = 65.0° for *f* = 100kHz, in contrast to the expected value of *θ* = 90°. Furthermore, the capacitance was observed to be independent of the frequency. Positive discharge exhibited a more stable current waveform than negative discharge. Mechanical vibration of the wire was minimised by fixing the wire with small pieces of dielectric (thin pieces of paper) inserted through the four gaps between neighbouring pairs of pipes. In the experiments, the current in the central pipe was measured for positive corona discharge with phase compensation of 5.8° for *f* = 20 kHz (see Fig. 4b) and 25.0° for *f* = 100kHz (see Fig. 4c).

The waveform *V*_*a*_ was generated using a high-voltage amplifier (10/40A, TREK) triggered by a function generator (WF1974, NF). *V*_*a*_ was measured using a 1000:1 divider inside the 10/40A amplifier. The circuit current *I* was calculated using Ohm’s law from the measured voltage drop across the shunt resistance *R*_*s*_. The waveforms of *V*_*a*_ and *I* were observed using a storage oscilloscope (6050A, LeCroy), and the stored data were processed to obtain the experimental data. The experiments were carried out with a temperature in the range 27.8-28.1 °C and a relative humidity in the range 51.0-52.2%.

### Simulation

We solved the Poisson’s equation (i.e., ∇^2^*V* = −*q*/*ε*) coupled with the charge conservation equation ∂*q*/∂*t* + ∇ · **J**_*f*_ = 0 for the space between *r*_*e*_ and *r*_*c*_, corresponding to the experimental arrangement. The Laplace equation ∇^2^*V* = 0 was also solved to obtain the electric field **E**_*Lp*_, which is necessary to calculate *I*_*S*_ and *I*_*SR*_. The term **J**_*f*_ in the charge conservation equation is given by **J**_*f*_ = *q*(**U**_*c*_ + **U**) −*D*∇*q*, where **U**_*c*_ is the convective fluid velocity, **U**( = *μ***E**) is the velocity of the charge (i.e., ion), *μ* is the mobility of the ion, and *D* is the diffusion coefficient^29^. The effects of **U**_*c*_ and diffusion were neglected (i.e., we assumed **U**_*c*_ = 0 and *D* = 0)^29^. We used a value of *μ* = 1.4 × 10^−4^ m^2^V^−1^s^−1^ for the mobility of the positive ions^35^,^36^. The permittivity of free space was used for *ε*^29^.

We followed the simulation method that we have previously reported for steady-state simulations^27^,^29^,^34^. To achieve the precise time-varying simulation required for this study, we took advantage of the symmetry of the system, so that the equations could be solved in one dimension, which provides significant gains in terms of the computational expense. *V*_*r*_ and *q*_*r*_ (see Fig. 2a) are functions of the radius and time. The grid structure was composed of one string of 236 cells with dense grids on the emitter side (start size: 2 μm, growth rate: 1.05, maximum size: 100 μm) to describe the large gradient of *V*_*r*_ and *q*_*r*_ near the emitter. The time-step was 0.2 ns, which maintains a maximum Courant number (charge velocity×iteration time/cell size) of Cr < 0.1 in the first cell above the surface of the emitter. Dirichlet boundary conditions were used for *V*_*r*_ at the emitter (*V*_*r*_ = *V*_*a*_) and the collector (*V*_*r*_ = 0), and we imposed ∂*q*_*r*_/∂*r* = 0 at both electrodes. A “constant charge density” (*q*_*i*_) model was used to represent the charge generation process in the plasma region around the emitter with the assumption that the radius of the plasma sheath is given by *r*_*p*_ ≈ 3*r*_*e*_^29^ (see the dotted circle in Fig. 2a). As the first step in the *q*_*i*_ decision process, we measured the DC current *I* for a given *V*_*a*_. The steady-state simulation subsequently followed to make the simulated current (i.e., integration of **J**_*f*_ on the collector surface) coincide with the measured *I* by adjusting *q*_*i*_ of the plasma region, and resulted in *q*_*i*_ = 0.0191 Cm^−3^ for *V*_*m*_ = 9.0 kV case and *q*_*i*_ = 0.0038 Cm^−3^ for *V*_*m*_ = 7.0 kV case. We used a *V*_*a*_ waveform with a very small ratio *V*_*o*_/*V*_*m*_ for the above plasma region model in the time-varying simulation, with a high frequency to increase the influence of capacitive currents *I*_*L*_ and *I*_*Q*_, i.e., *V*_*o*_/*V*_*m*_ = 0.059 (0.53 kV and 9.0 kV) at 20 kHz and *V*_*o*_/*V*_*m*_ = 0.0071 (0.05 kV and 7.0 kV) at 100 kHz.

From the simulated time-varying *V* and *q* fields, we calculated the components of the currents shown in Fig. 4a using Δ*K*_*X*_/Δ*t*/*V*_*a*_ (where Δ*t* = 40 ns); 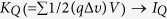, 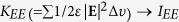, 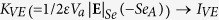, 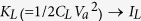, 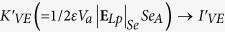, 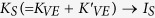 and 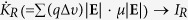, where Δ*υ* is the volume of each cell, and *Se*_*A*_ is the area of the surface of the emitter. The circuit components shown in Fig. 5 were calculated using 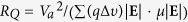, 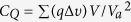, 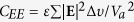 and 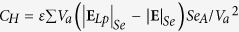.

## Additional Information

**How to cite this article**: Kim, C. *et al.* Circuital characterisation of space-charge motion with a time-varying applied bias. *Sci. Rep.* **5**, 11738; doi: 10.1038/srep11738 (2015).
